# miR-223 overexpression inhibits doxorubicin-induced autophagy by targeting FOXO3a and reverses chemoresistance in hepatocellular carcinoma cells

**DOI:** 10.1038/s41419-019-2053-8

**Published:** 2019-11-06

**Authors:** Yue Zhou, Enjiang Chen, Yuexiao Tang, Jiayan Mao, Jian Shen, Xiaoxiao Zheng, Shangzhi Xie, Shufen Zhang, Ying Wu, Hao Liu, Xiao Zhi, Tao Ma, Haibin Ni, Jiabin Chen, Kequn Chai, Wei Chen

**Affiliations:** 1 Department of Hepatobiliary and Pancreatic Surgery, The First Affiliated Hospital of Zhejiang University, School of Medicine, 310009 Hangzhou, Zhejiang China; 2 The Second Affiliated Hospital of Zhejiang University, School of Medicine, 310009 Hangzhou, Zhejiang China; 3 Department of Genetics, Research Center for Molecular Medicine, Institute of Cell Biology, Key Laboratory of Reproductive Genetics, Ministry of Education, Zhejiang University School of Medicine, 310058 Hangzhou, Zhejiang China; 4 Cancer Institute of Integrated traditional Chinese and Western Medicine, Key laboratory of cancer prevention and therapy combining traditional Chinese and Western Medicine, Zhejiang Academy of Traditional Chinese Medicine, 310012 Hangzhou, Zhejiang China; 5 Department of Surgery, Anschutz Medical Campus, University of Colorado, Aurora, CO 80045 USA; 6 Department of Gastrointestinal Surgery, Tongde Hospital of Zhejiang Province, 310012 Hangzhou, Zhejiang China; 7 Department of Medical Oncology, Tongde Hospital of Zhejiang Province, 310012 Hangzhou, Zhejiang China

**Keywords:** Cancer therapeutic resistance, Chemotherapy

## Abstract

Doxorubicin is conventionally used in chemotherapy against hepatocellular carcinoma (HCC), but acquired resistance developed during long-term therapy limits its benefits. Autophagy, a conserved catabolic process for cellular self-protection and adaptation to the changing environment, is regarded as a potential clinical target to overcome doxorubicin resistance. In this study, the potential role of miR-223 in modulating doxorubicin-induced autophagy and sensitivity were evaluated in four transfected human HCC cell lines, and the in vivo relevance was assessed using a mouse xenograft model of HCC. We found that the well-defined miR-223 is expressed at low levels in doxorubicin treated HCC cells and that miR-223 overexpression inhibits the doxorubicin-induced autophagy that contributes to chemoresistance. Blockade of autophagic flux by chloroquine resulted in the failure of miR-223 inhibitor to suppress doxorubicin sensitivity of HCC cells. We further identified FOXO3a as a direct downstream target of miR-223 and primary mediator of the regulatory effect of miR-223 on doxorubicin-induced autophagy and chemoresistance in HCC cells. Finally, we confirmed the enhancement of doxorubicin sensitivity by agomiR-223 in xenograft models of HCC. These findings establish a novel miRNA-based approach for autophagy interference to reverse doxorubicin resistance in future chemotherapy regimens against human HCC.

## Introduction

Hepatocellular carcinoma (HCC) is one of the most common and deadliest malignancies worldwide^1^. Doxorubicin has been widely used in systemic and local anti-HCC therapy, and still remains the first-line agent for chemoembolization of HCC today^2^. However, acquired resistance developed during long-term chemotherapy severely compromises its therapeutic benefits for this fatal disease^3^. Thus, novel advanced strategies to improve drug response and reduce side effects of doxorubicin are needed. With better understanding over the last decade of the molecular mechanism for chemoresistance, rational combination of targeted agents with traditional doxorubicin is regarded as a promising approach in HCC treatment^4–6^.

Autophagy is a highly conserved catabolic process induced by various cellular stresses including energy or nutrient shortage and cytotoxic insults, and performs the primary functions of cellular self-protection and adaptation to the changing environment^7^. Doxorubicin treatment induces autophagy which contributes to the development of chemoresistance, and inhibition of autophagy effectively overcomes or reverses doxorubicin resistance in a variety of cancers^8–10^. Although a number of autophagy-targeted interventions such as Lys05, HSF1/ATG4B knockdown, and ADCX have been reported to sensitize HCC cells to doxorubicin^11–13^, clinically beneficial autophagy modulations against doxorubicin resistance in HCC patients are still rare and need further exploration.

MicroRNAs (miRNAs), endogenous non-coding RNAs that cause translational inhibition or degrade target mRNAs, have shown enormous clinical potential in various liver diseases^14^. Increasing evidence demonstrates that several miRNAs are also implicated in doxorubicin resistance and are promising targets for combined treatment of HCC^15–17^. miR-223, a commonly repressed miRNA in HCC cells, has been confirmed to be involved in many important physiological and pathological processes including proliferation, metastasis, and stemness maintenance in HCC, while miR-223 targeted therapy has good prospect for clinical application^18–21^. Previous studies also reveal that miR-223 regulates the multidrug resistance of HCC cells^22,23^. In addition, recent research indicates that miR-223 suppresses excessive autophagy in cardiomyocytes^24^. Nevertheless, whether miR-223 can modulate doxorubicin-induced autophagy in HCC cells remains unclear.

FOXO3a, a multifaceted transcription factor that integrates cellular and environmental stresses^25^, is widely accepted to guide autophagy directly or indirectly^26–28^. Recent research demonstrates that FOXO3a is also involved in doxorubicin-induced autophagy^10,29^. Meanwhile, FOXO3a expression is reported to be suppressed by miR-223 in multiple diseases^30–32^. Furthermore, FOXO3a participates in the regulation of doxorubicin resistance in HCC^33^. Taken together, miR-223 might modulate autophagy via FOXO3a in HCC cells. We report herein the role of miR-223 in autophagy regulation in doxorubicin-treated HCC cells. Our results demonstrate that upregulating miR-223 could suppress doxorubicin-induced autophagy, thereby enhancing doxorubicin cytotoxicity in HCC cells. Moreover, we define FOXO3a as a critical downstream target of miR-223 to govern the autophagic activity of HCC cells.

## Materials and methods

### Cell lines and cultures

Human HCC cell lines (HepG2, Huh7, SNU387, and SNU449) and human embryonic kidney cell line (HEK-293T) were purchased from the American Type Culture Collection (ATCC; Manassas, VA, USA). Huh7 and HepG2 cells were cultured in high glucose DMEM (Gibco; Carlsbad, CA, USA) containing 10% fetal bovine serum (FBS; Gibco) and 1% penicillin/streptomycin (Sigma-Aldrich; St. Louis, MO, USA). SNU449, SNU387, and 293T cells were cultured in RPMI Medium (Gibco) supplemented with 10% FBS and 1% penicillin/streptomycin. Cells were maintained at 37 °C in 5% CO_2_ and 95% air. All cell lines were authenticated using STR DNA fingerprinting (Shanghai Biowing Applied Biotechnology Co.; Shanghai, China), and mycoplasma infection was detected using LookOut Mycoplasma PCR Detection Kit (Sigma-Aldrich).

### Drugs and antibodies

Doxorubicin and chloroquine were purchased from Sigma-Aldrich. AgomiR-223 and the negative control (NC agomiR) were purchased from RiboBio Co. (Guangzhou, China). LC3, p62, FOXO3a, and GAPDH primary antibodies and anti-rabbit IgG HRP-linked secondary antibody for Western blotting were obtained from Cell Signaling Technology (CST; Danvers, MA, USA).

### Transfection assay

Synthesized miR-223 mimic, miR-223 inhibitor, and the respective negative controls (NC mimic and NC inhibitor) were purchased from RiboBio, and synthesized FOXO3a siRNA and the negative control (NC siRNA) were purchased from GenePharma Co. (Shanghai, China), with detailed sequences listed in Supplementary Table S1. HCC cells were transfected for 6 h with siRNA, inhibitor, or mimic using X-tremeGENE Transfection Reagent (Roche; Indianapolis, IN, USA) following the manufacturer’s instructions. Opti-MEM transfection medium (Gibco) was replaced with complete medium, and the transfection efficacy was assessed. All subsequent experiments were performed 72 h after transfection.

### Quantitative real-time polymerase chain reaction (qRT-PCR)

Total RNA (miRNA and mRNA) was extracted using TRIzol Reagent (Invitrogen; Carlsbad, CA, USA). Reverse transcription of miRNA and mRNA was performed using Mir-X miRNA First-Strand Synthesis Kit and PrimeScript RT Reagent Kit (Takara; Dalian, China), respectively, according to the manufacturer’s protocol. Primers for miR-223**-**3p, U6, FOXO3a, and GAPDH were designed and purchased from Takara with detailed sequences listed in Supplementary Table S2. Quantitative PCR was performed using SYBR Premix EX Taq Kit (Takara) on an ABI Prism 7900HT Real-Time System (Applied Biosystems Inc; Shanghai, China) followed by melting curve analysis. All experiments were carried out in triplicate and independently repeated three times. MiR-223 and FOXO3a mRNA expression were normalized to U6 small nuclear RNA and GAPDH, respectively, and analyzed according to the 2^−ΔΔCt^ method.

### Western blot assay

HCC cells washed with PBS were resuspended in cell lysis buffer (CST), and protein concentration was quantified using the BCA protein assay kit (ThermoFisher; Rockford, IL, USA) according to the manufacturer’s protocol. Prepared protein lysates mixed with NuPage loading buffer (Invitrogen) were then denatured and separated through 10% SDS-PAGE gel electrophoresis, followed by transfer to PVDF membranes (Millipore; Billerica, MA, USA). After antibody incubation and chemiluminescence detection (GE Healthcare; Piscataway, NJ, USA), protein bands were visualized using a ChemiDoc XRS Imaging System (Bio-Rad; Hercules, CA, USA). Band densities were estimated by Image Pro Plus (Media Cybernetics, Inc.; Bethesda, MD, USA), while relative protein expression levels were normalized to GAPDH.

### Cell viability assay

Normal or preconditioned HCC cells were seeded into 96-well microplates and incubated overnight. Culture medium was then replaced with media containing different concentrations of doxorubicin (0, 0.0625, 0.125, 0.25, 0.5, and 1 μg/ml) or chloroquine (20 μM) for 48 h. Cell viability was assessed using a cell counting kit-8 assay (CCK-8; Dojindo; Kumamoto, Japan) according to the manufacturer’s protocol. Half maximal inhibitory concentration (IC50) of doxorubicin was calculated by fitting data to the equation: V% = 100/(1 + 10^[Drug]log IC50^), where V% is the percentage viability and [Drug] is the doxorubicin concentration (μg/ml). All experiments were carried out with five duplications and independently repeated three times.

### LC3-dual-fluorescence assay

HCC cells seeded on microscope cover glass were transfected with the mRFP-GFP-LC3 adenovirus (Hanbio; Shanghai, China) for 24 h. Then cells were transfected with miR-223 mimic, miR-223 inhibitor, or FOXO3a siRNA, and treated with doxorubicin (0.25 μg/ml) for 48 h. After the above procedure, cells were fixed with 4% paraformaldehyde, and images were obtained using a TCS SP8 MP laser scanning confocal microscope (Leica; Wetzlar, Germany). Autophagy flux was then assessed by confocal counting of the GFP positive/mRFP positive (yellow) and GFP negative/mRFP positive (red) puncta in cells. 50–100 cells were counted per group in triplicate experiments.

### TEM analysis

HCC cells preconditioned for 48 h were harvested. After trypsinization and rinsing twice, cells were fixed in 2.5% glutaraldehyde and 0.1 M cacodylate buffer for 2 h. After washing and post-fixation in 1% osmium tetroxide and 0.1 M cacodylate buffer, cells were again washed and then dehydrated through a graded series of ethanol solutions, and finally embedded in epoxy resin. Ultrastructures of autophagy in cells were observed and imaged under a Tecnai transmission electron microscope (FEI; Hillsboro, OR, USA) at an operating voltage of 80 kV.

### Luciferase reporter assay

The 3′-UTR fragment of FOXO3a was amplified and cloned into the psiCHECK2 vector (Promega; Madison, WI, USA). The binding site for miR-223 was identified using TargetScan algorithm and mutated with QuikChange Multi Site-Directed Mutagenesis Kit (Agilent Technologies Inc.; Santa Clara, CA, USA). The resulting plasmids were sequenced and then co-transfected with NC mimic or miR-223 mimic into HEK-293T cells using FuGENE HD Transfection Reagent (Promega). Cells were lysed after 24 h and luciferase expression was measured using the Dual-Glo luciferase assay system (Promega) following the manufacturer’s instructions. All experiments were performed in triplicate and the renilla luciferase activity was normalized to that of firefly luciferase.

### In vivo tumor model

Animal experiments were conducted in compliance with the Guide for the Care and Use of Animal Ethics Committee of Zhejiang University (Hangzhou, China). 20 male nude mice (Shanghai Experiment Animal Centre; Shanghai, China) were raised with irradiated fodder under specific pathogen free conditions and randomly subdivided into four subgroups. 1 × 10^6^ prepared Huh-7 cells were injected subcutaneously into the right axillary fossa. Tumor length (L) and width (W) were measured and tumor volumes were calculated using the formula (L × W^2^)/2. When tumor volumes reached 50–100 mm^3^, drug treatment was initiated as follows: negative control, 4 mg/kg doxorubicin, 3 nmol agomiR-223, or doxorubicin combined with agomiR-223. Doxorubicin and agomiR-223 were administered by intraperitoneal and intratumor injection, respectively, every 2 days for 2 weeks. Mice were subsequently euthanized by cervical dislocation and tumors were dissected.

### Statistical analyses

Independent experiments were performed in triplicate, and all experimental data are expressed as the mean and standard deviation (SD). Statistical analysis was carried out using Prism 5 (Version 6.0; GraphPad; San Diego, CA, USA). Statistical significance was defined as a *p*-value < 0.05, assessed by one-way analysis of variance (ANOVA) with Bonferroni correction.

## Results

### miR-223 overexpression sensitizes HCC cells to doxorubicin in vitro

**Fig. 1:**
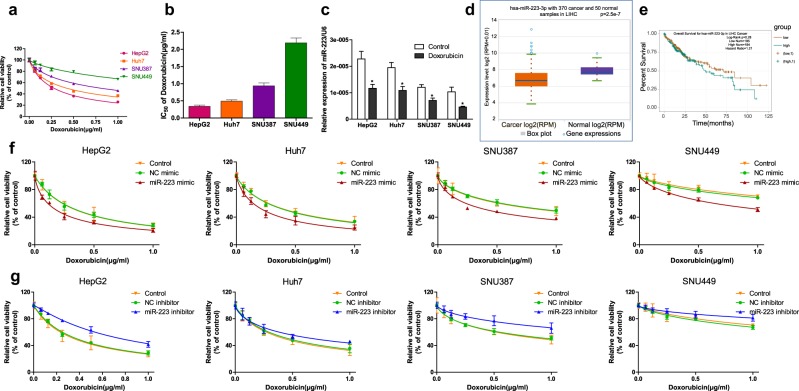
miR-223 overexpression sensitizes HCC cells to doxorubicin in vitro. **a** Relative cell viability (mean ± SD) for HepG2, HuH-7, SNU-387, and SNU-449 cells treated with doxorubicin for 48 h. **b** IC50s of doxorubicin (mean ± SD) in different HCC cells. **c** Relative miR-223 expression in untreated control or doxorubicin treated HCC cells. Data were normalized to levels of U6 (*p < 0.05, Control vs. Doxorubicin). **d** miR-223 expression level of 370 HCC and 50 normal samples (*p* = 2.5e−7, Cancer samples vs. Normal samples). **e** Overall survival of HCC patients with high or low miR-223 expression levels estimated using the Kaplan–Meier method and compared by the log-rank test (*p* = 0.28, low miR-223 expression vs. high miR-223 expression; Hazard Ratio = 1.21). **f**, **g** Relative cell viability (mean ± SD) for miR-223 mimic or inhibitor transfected HepG2, HuH-7, SNU-387, and SNU-449 cells treated with doxorubicin for 48 h

Doxorubicin cytotoxicity for HepG2, Huh7, SNU387, and SNU449 cell lines was examined. HepG2 cells were found to be the most sensitive to doxorubicin, whereas SNU449 cells possessed the least sensitivity accompanied with the highest IC50 (Fig. 1a, b). We then detected miR-223 expression in different HCC cell lines. Interestingly, cell lines with higher doxorubicin sensitivity exhibited higher miR-223 expression; HepG2 and SNU449 cells expressed the highest and lowest miR-223 levels, respectively. Moreover, miR-223 expression in doxorubicin-treated HCC cells was significantly decreased compared to untreated control (Fig. 1c). In addition, analysis results from starBase v3.0 (http://starbase.sysu.edu.cn/) database^34^ showed that miR-223 expression levels were significantly lower in HCC samples compared with normal samples, while there was no significant correlation between miR-223 expression and overall survival of HCC patients (Fig. 1d, e).

To further investigate the role of miR-223 in regulating doxorubicin sensitivity of HCC cells, doxorubicin cytotoxicity was measured following miR-223 transfection in different HCC cell lines. Transfection efficiency of miR-223 mimic or inhibitor was evaluated beforehand (Fig. S1a, b). As shown in Fig. 1f, g, doxorubicin cytotoxicity for the miR-223 mimic transfected HCC cells was increased compared with the negative control, while miR-223 inhibitor transfection led to reduced doxorubicin sensitivity of HCC cells. IC50 values were then calculated based on the dose-effect curves. Compared to the negative control, doxorubicin IC50s in miR-223 mimic transfected HCC cells were remarkably lower, whereas conversely the IC50s in miR-223 inhibitor transfected HCC cells were higher, suggesting that miR-223 overexpression sensitized HCC cells to doxorubicin (Fig. S1c, d).

### Autophagy inhibition potentiates doxorubicin sensitivity of HCC cells

**Fig. 2:**
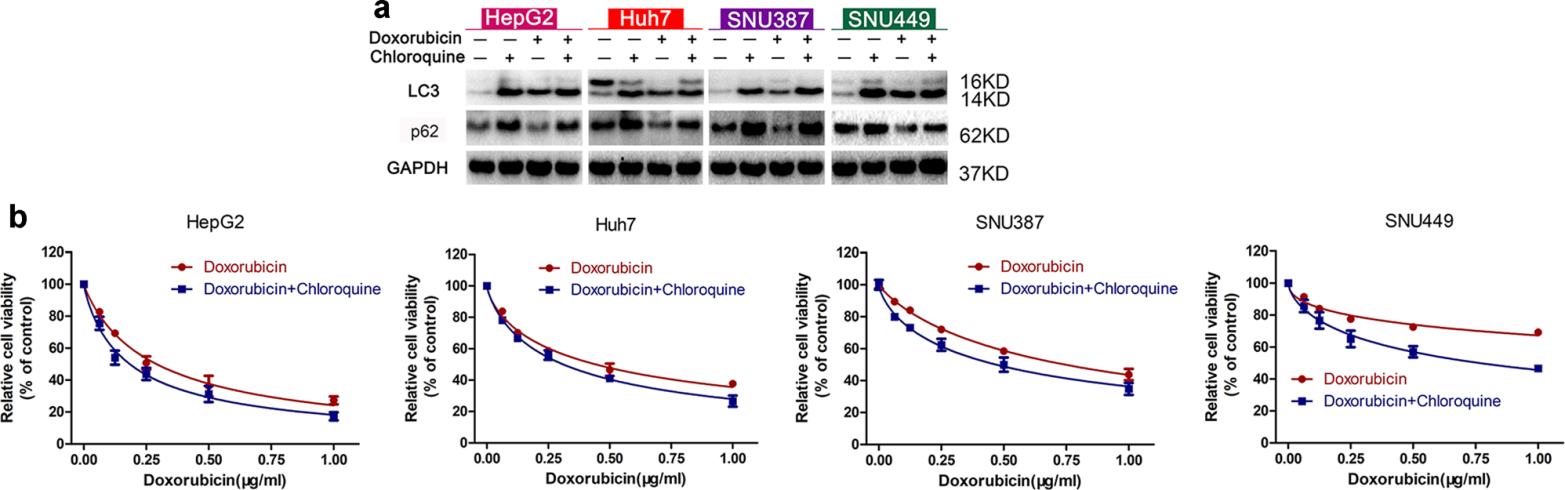
Autophagy inhibition potentiates doxorubicin sensitivity for HCC cells. **a** LC3 and p62 protein expression in control HCC cells and HCC cells treated for 48 h with chloroquine (20 μM), doxorubicin (0.25 μg/ml), or doxorubicin plus chloroquine. **b** Relative cell viability for HepG2, HuH-7, SNU-387, and SNU-449 cells treated with doxorubicin in the presence or absence of chloroquine for 48 h

To verify the influence of inducible autophagy on doxorubicin sensitivity of HCC cells, the classic autophagy inhibitor chloroquine was used to prevent active autophagy. Doxorubicin treatment led to a notably increased ratio of LC3-II/LC3-I and decreased p62 protein expression in all four HCC cell lines examined. While combined treatment with chloroquine, which exhibited almost no cytotoxicity in HCC cells at the concentration of 20 μM (Fig. S2a), further elevated LC3-II/LC3-I ratio and reversely increased p62 protein expression obviously (Fig. 2a), indicating that chloroquine prevented the doxorubicin-induced autophagy. We next examined doxorubicin cytotoxicity for HepG2, Huh7, SNU387, and SNU449 cell lines in the presence or absence of chloroquine. As shown in Fig. 2b, compared with doxorubicin alone, combined chloroquine and doxorubicin treatment exerted enhanced cytotoxicity for all four HCC cells. Meanwhile, IC50 calculation demonstrated that in comparison with doxorubicin alone, doxorubicin IC50s in the presence of chloroquine were remarkably diminished (Fig. S2b). These data confirm that autophagy inhibition potentiates doxorubicin sensitivity for HCC cells.

### miR-223 intervention modulates doxorubicin-induced autophagy in HCC cells

**Fig. 3:**
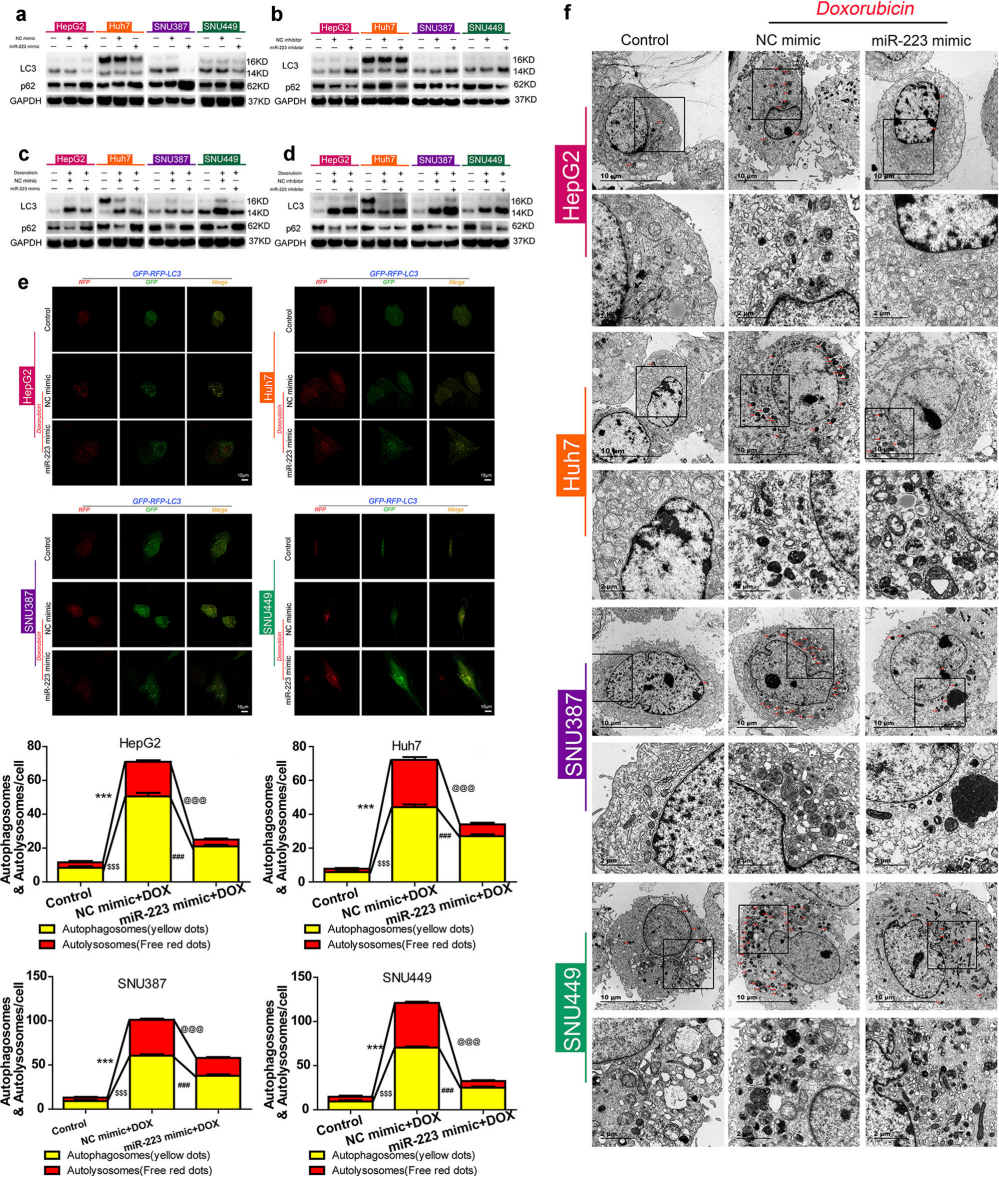
miR-223 intervention modulates doxorubicin-induced autophagy in HCC cells. **a**, **b** LC3 and p62 protein expression in control HCC cells and HCC cells transfected with NC mimic, miR-223 mimic, NC inhibitor, or miR-223 inhibitor for 48 h. **c**, **d** LC3 and p62 protein expression in control HCC cells and HCC cells transfected with NC mimic, miR-223 mimic, NC inhibitor, or miR-223 inhibitor followed by doxorubicin treatment for 48 h. **e** Up, mRFP-GFP-LC3 stable HepG2, HuH-7, SNU-387, and SNU-449 cells in the untreated groups or groups with NC mimic or miR-223 mimic transfection followed by doxorubicin treatment for 48 h were visualized by confocal microscopy. Down, number of GFP^+^/mRFP^+^-LC3 (yellow) and GFP^−^/mRFP^+^-LC3 (red) dots were scored on 50 cells. (^$$$^*p* < 0.001, ***p < 0.001, Control vs. NC mimics plus doxorubicin treatment; ^@@@^*p* < 0.001, ^###^*p* < 0.001, NC mimic plus doxorubicin treatment vs. miR-223 mimic plus doxorubicin treatment). **f** Ultrastructural features of intracellular double-membrane vesicles control HCC cells and HCC cells transfected with NC mimic or miR-223 mimic followed by doxorubicin treatment counterstained with 4% uranyl acetate, as observed by TEM. Scale bar = 10 μm

To explore whether miR-223 is involved in autophagy of HCC cells, LC3 and p62 protein expression was detected after miR-223 intervention. Compared with the negative control, miR-223 mimic transfection of HCC cells resulted in a decreased LC3-II/LC3-I ratio and increased p62 protein expression, while transfection of miR-223 inhibitor led to opposite results (Fig. 3a, b).

As reported above, doxorubicin treatment triggered autophagy and elicited low expression of miR-223 in HCC cells, and miR-223 was shown to suppress autophagy in HCC cells. We therefore investigated the effect of miR-223 intervention on doxorubicin-induced autophagy. For both miR-223 mimic and inhibitor transfected HCC cells, autophagic activity was estimated after doxorubicin treatment for 48 h. In the negative control groups, doxorubicin treatment raised the LC3-II/LC3-I ratio and decreased p62 protein expression. In comparison, doxorubicin treatment in miR-223-overexpressed HCC cells failed to induce a significant increase in the LC3-II/LC3-I ratio or a decrease in p62 expression (Fig. 3c); whereas in HCC cells transfected with miR-223 inhibitor, doxorubicin-induced autophagy was dramatically enhanced (Fig. 3d). Meanwhile, LC3-dual-fluorescence assay also revealed that a few punctate fluorescent patterns were observed in the untreated control cells, identifying the autophagosomes (yellow dots) and autolysosomes (red dots), whereas doxorubicin-treated HCC cells exhibited a striking accumulation of punctate fluorescent patterns. In contrast with NC mimic groups, doxorubicin treatment failed to cause a similarly notable accumulation of punctate fluorescent patterns in miR-223 overexpressed HCC cells (Fig. 3e). Compared with NC inhibitor groups, doxorubicin treatment triggered a remarkable increase in autolysosomes in HCC cells transfected with miR-223 inhibitor (Fig. S3). Furthermore, morphology observation by TEM clearly demonstrated a greater number of autophagosomes with typical double-layer membranes containing organelle remnants in doxorubicin-treated HCC cells. Compared with the negative control groups treated with doxorubicin, the miR-223 mimic or inhibitor transfection groups were consistent with Western blot and LC3-dual-fluorescence assay findings (Figs. 3f and S4). Taken together, these data demonstrate that miR-223 plays a crucial role in modulating doxorubicin-induced autophagy in HCC cells.

### miR-223 influences doxorubicin resistance of HCC cells via regulation of autophagy

**Fig. 4:**
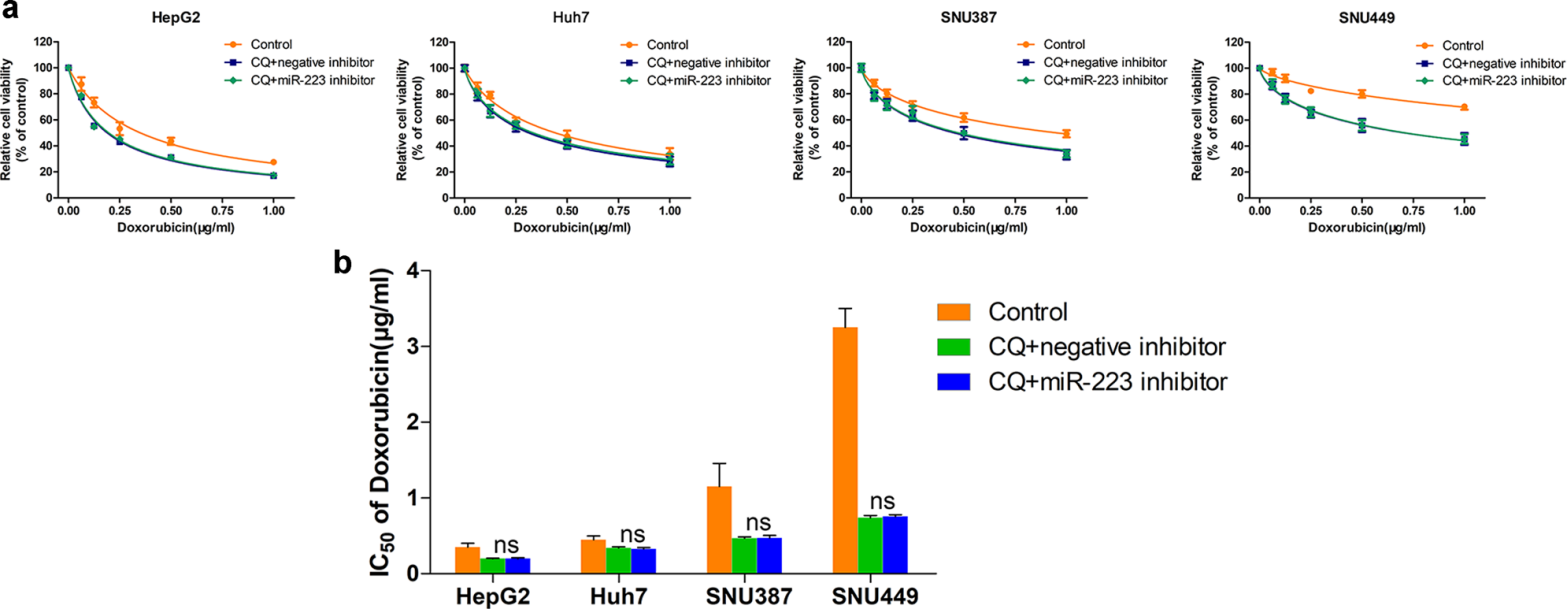
miR-223 influences doxorubicin resistance of HCC cells via regulation of autophagy. **a** Relative cell viability (mean ± SD) for chloroquine-treated and miR-223 or NC inhibitor transfected HepG2, HuH-7, SNU-387, and SNU-449 cells treated with doxorubicin for 48 h. **b** IC50 values of doxorubicin in chloroquine-treated and miR-223 or NC inhibitor transfected HCC cells (n.s. *p* > 0.05, miR-223 inhibitor plus chloroquine vs. NC inhibitor plus chloroquine)

Because our data demonstrated that miR-223 overexpression inhibited doxorubicin-induced autophagy and led to reduced doxorubicin resistance for HCC cells, while autophagy inhibition promoted doxorubicin sensitivity, we further explored the role of autophagy in miR-223 regulated doxorubicin resistance. Doxorubicin cytotoxicity was detected in chloroquine-treated HCC cell lines followed by miR-223 inhibitor transfection. In all four chloroquine-treated HCC cell lines with notably blocked autophagy flux, no significant difference of doxorubicin sensitivity could be found between the miR-223 inhibitor group and the negative control group (Fig. 4a, b), indicating that miR-223 influences doxorubicin-resistance of HCC cells via regulation of autophagy.

### FOXO3a is a direct downstream target of miR-223 in HCC cells

**Fig. 5:**
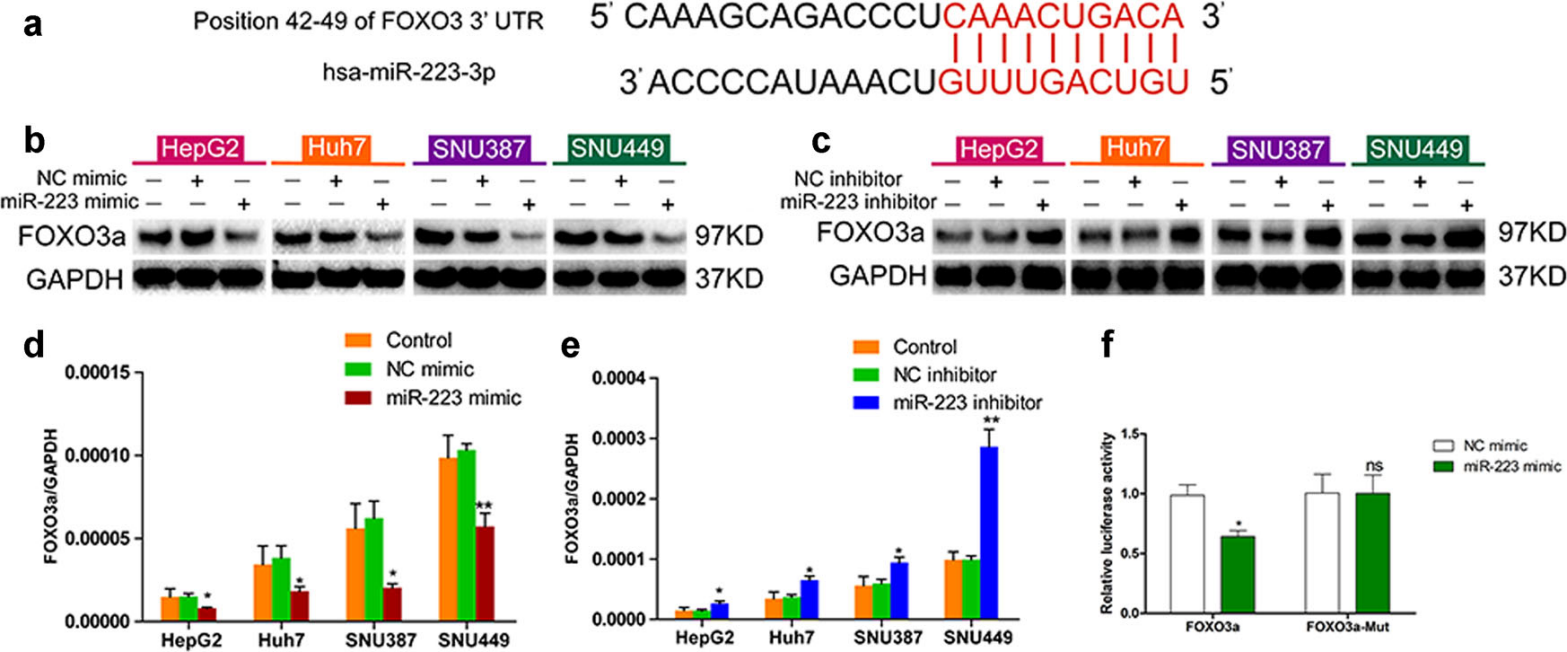
FOXO3a is a downstream target of miR-223 in HCC cells. **a** Prediction of miR-223 targets in TargetScan. Predicted consequential pairing of target region in the FOXO3a 3’-UTR (top) and has-miR-223-3p (bottom). **b**, **c** FOXO3a protein expression in control HCC cells and HCC cells transfected with NC mimic, miR-223 mimic, NC inhibitor, or miR-223 inhibitor for 48 h. **d**, **e** FOXO3a mRNA expression in control HCC cells and HCC cells transfected with NC mimic, miR-223 mimic, NC inhibitor, or miR-223 inhibitor for 48 h (*p < 0.05, **p < 0.01, miR-223 mimic vs. NC mimic or miR-223 inhibitor vs. NC inhibitor). **f** Effect of NC mimic or miR-223 mimic on luciferase activity in 293T cells transfected with either the wild type or mutant FOXO3a 3’-UTR psiCHECK2 plasmid (*p < 0.05, miR-223 mimic vs. NC mimic)

To further elucidate the mechanism of miR-223 in autophagy modulation, target prediction was performed using TargetScan and FOXO3a was identify as a candidate miR-223 target (Fig. 5a). Subsequently, FOXO3a mRNA and protein expression in miR-223 inhibitor or mimic transfected HCC cells were measured to confirm the prediction. We found that FOXO3a expression was significantly increased in the miR-223 inhibitor group compared with the NC inhibitor group while miR-223 mimic transfection caused markedly decreased FOXO3a expression (Fig. 5b–e), suggesting that FOXO3a is a downstream target of miR-223 in HCC cells. To investigate whether FOXO3a is a direct target regulated by miR-223, we performed luciferase reporter assay in 293T cells. In cells transfected with a FOXO3a 3’-UTR psiCHECK2 plasmid, miR-223 mimic co-transfection resulted in significantly lower luciferase activity compared with the NC mimic group. No notable difference in luciferase activity was detected between the miR-223 mimic and NC mimic groups in cells transfected with a mutant FOXO3a 3’-UTR psiCHECK2 plasmid, indicating that miR-223 directly regulates FOXO3a expression (Fig. 5f).

### FOXO3a plays a critical role in doxorubicin-induced autophagy of HCC cells

**Fig. 6:**
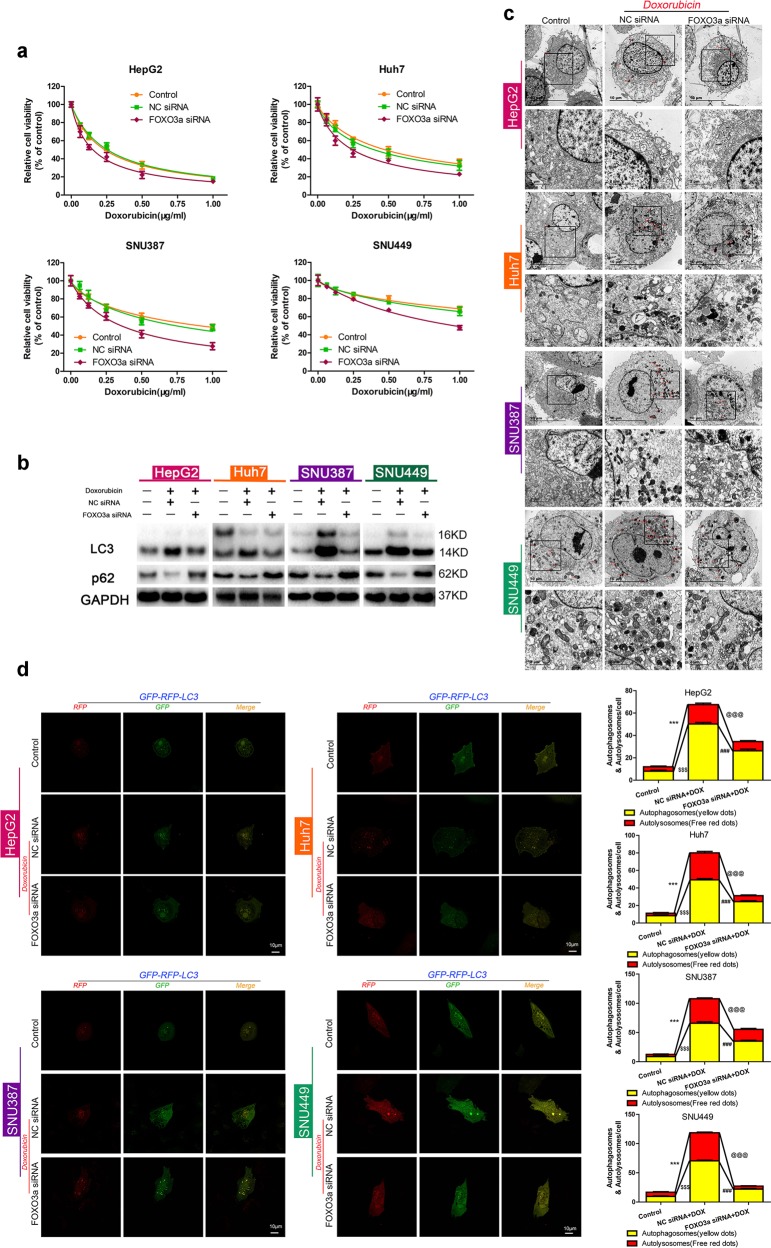
FOXO3a plays critical roles in doxorubicin-induced autophagy and chemosensitivity of HCC cells. **a** Relative cell viability (mean ± SD) for FOXO3a or NC siRNA transfected HepG2, HuH-7, SNU-387, and SNU-449 cells treated with doxorubicin for 48 h. **b** LC3 and p62 protein expression in control HCC cells and HCC cells transfected with FOXO3a siRNA or NC siRNA followed by doxorubicin treatment for 48 h. **c** Ultrastructural features of intracellular double-membrane vesicles in control HCC cells and HCC cells transfected with FOXO3a siRNA or NC siRNA followed by doxorubicin treatment, counterstained with 4% uranyl acetate and observed by TEM. Scale bar = 10 μm. **d** Up, mRFP-GFP-LC3 stable HepG2, HuH-7, SNU-387, and SNU-449 cells in the untreated groups or groups with FOXO3a siRNA or NC siRNA transfection followed by doxorubicin treatment for 48 h were visualized by confocal microscopy. Down, number of GFP^+^/mRFP^+^-LC3 (yellow) and GFP^−^/mRFP^+^-LC3 (red) dots were scored on 50 cells. (^$$$^*p* < 0.001, ***p < 0.001, Control vs. NC siRNA plus doxorubicin treatment; ^@@@^*p* < 0.001, ^###^*p* < 0.001, NC siRNA plus doxorubicin treatment vs. FOXO3a siRNA plus doxorubicin treatment)

To investigate whether FOXO3a is essential in regulating doxorubicin induced autophagy and resistance in HCC, doxorubicin cytotoxicity and autophagic activity were assessed following FOXO3a knockdown. Transfection efficacy of FOXO3a siRNA was checked first (Fig. S5a, b). Compared with the negative control, doxorubicin cytotoxicity in FOXO3a siRNA transfected HCC cells was dramatically enhanced (Fig. 6a). Meanwhile, the doxorubicin IC50 of HCC cells was significantly decreased after downregulation of FOXO3a (Fig. S5c). Furthermore, FOXO3a siRNA transfection led to remarkably decreased LC3-II/LC3-I ratio and increased p62 expression, and visually reduced red and yellow punctate fluorescent patterns, as well as decreased number of autophagosomes with typical double-layer membranes, in all four HCC cell types after treatment with doxorubicin for 48 h (Fig. 6b–d). Therefore, we conclude that FOXO3a plays critical roles in both doxorubicin-induced autophagy and doxorubicin resistance in HCC cells.

### miR-223 directs doxorubicin-induced autophagy in HCC cells by targeting FOXO3a

**Fig. 7:**
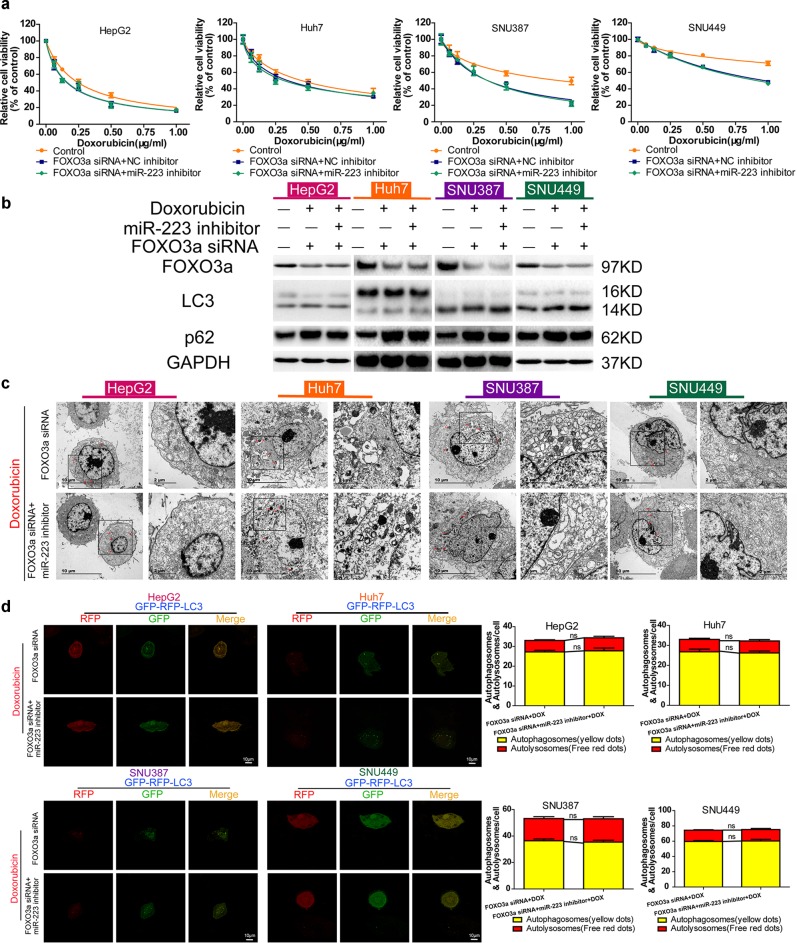
miR-223 directs doxorubicin-induced autophagy in HCC cells by targeting FOXO3a. **a** Relative cell viability (mean ± SD) for FOXO3a siRNA transfected HepG2, HuH-7, SNU-387 and SNU-449 cell lines, followed by miR-223 or NC inhibitor transfection and doxorubicin treatment for 48 h. **b** FOXO3a, LC3, and p62 protein expression in control HCC cells and FOXO3a siRNA transfected HCC cell lines, followed by miR-223 or NC inhibitor transfection and doxorubicin treatment for 48 h. **c** Ultrastructural features of intracellular double-membrane vesicles in FOXO3a siRNA transfected HCC cell lines followed by miR-223 or NC inhibitor transfection and doxorubicin treatment, counterstained with 4% uranyl acetate and observed by TEM. Scale bar = 10 μm. **d** Left, mRFP-GFP-LC3 stable HepG2, HuH-7, SNU-387, and SNU-449 cells in FOXO3a siRNA transfected groups followed by miR-223 or NC inhibitor transfection and doxorubicin treatment for 48 h were visualized by confocal microscopy. Right, number of GFP^+^/mRFP^+^-LC3 (yellow) and GFP^−^/mRFP^+^-LC3 (red) dots were scored on 50 cells. (n.s. *p* > 0.05, FOXO3a siRNA plus doxorubicin treatment vs. miR-223 inhibitor plus FOXO3a siRNA plus doxorubicin treatment)

Previous findings had demonstrated that miR-223 governed the autophagic activity of HCC cells, while as a direct target of miR-223, FOXO3a was essential in regulating doxorubicin-induced autophagy in HCC. Thus, we wondered whether FOXO3a mediated the regulatory effect of miR-223 on doxorubicin autophagy in HCC. Doxorubicin cytotoxicity was detected in FOXO3a siRNA transfected HCC cell lines followed by miR-223 or NC inhibitor transfection. In all four HCC cell lines, FOXO3a downregulation resulted in elevated cytotoxicity, whereas no significant difference in doxorubicin sensitivity was detected between the miR-223 inhibitor group and the negative control group (Fig. 7a). Moreover, compared with the negative control, miR-223 inhibitor failed to cause an increase in the LC3-II/LC3-I ratio and a decrease in p62 expression of HCC cells treated with doxorubicin for 48 h (Fig. 7b). The results of TEM detection and LC3-dual-fluorescence assay were consistent with the above observations (Fig. 7c, d). In addition, analysis results from starBase v3.0 database also showed that, contrary to the lower miR-223 expression, FOXO3a expression were higher in HCC samples compared with normal samples, although both the FOXO3a expression difference and its correlation with overall survival of HCC patients had no statistical significance (Fig. S6a, b). These data demonstrate that FOXO3a is a downstream target through which miR-223 influences doxorubicin-induced autophagy in HCC cells.

### miR-223 overexpression enhances the in vivo efficacy of doxorubicin for HCC

**Fig. 8:**
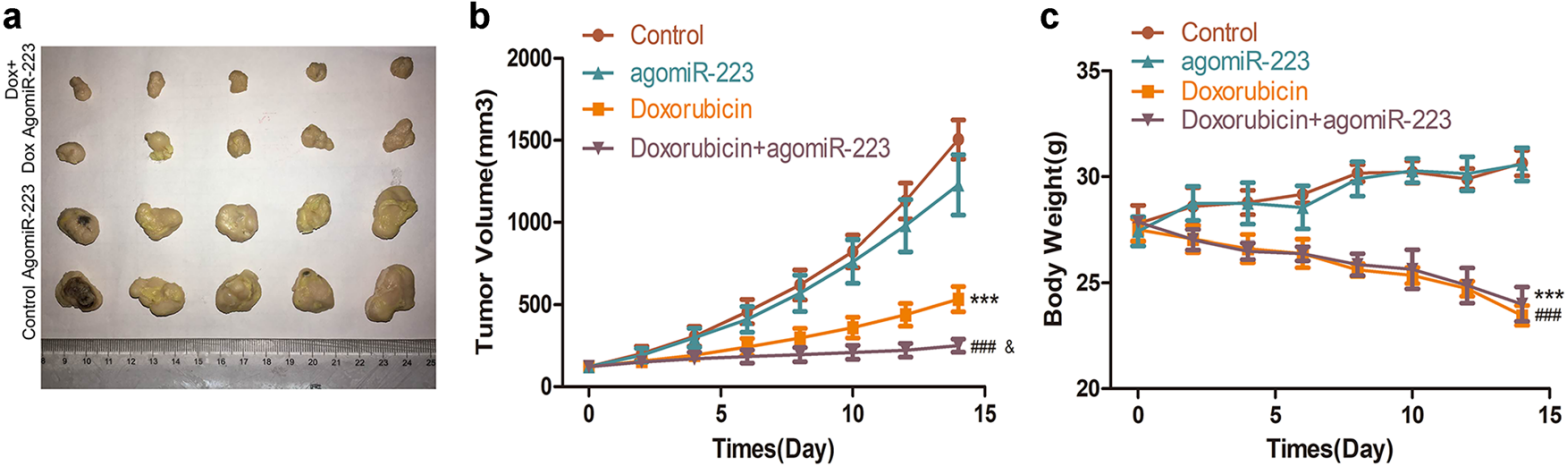
miR-223 overexpression enhances the in vivo efficacy of doxorubicin for HCC. **a** After 2 weeks of treatment, mice of different groups were euthanized and tumors were dissected. **b** Volume of tumor xenografts in the control (red), AgomiR-223 (orange), doxorubicin (green), or doxorubicin plus AgomiR-223 (violet) groups. Relative tumor volume ratios (% of original volume when therapy initiated) are presented as the mean ± SD, *n* = 5 (***p < 0.001, control vs. doxorubicin alone; ^###^*p* < 0.001, doxorubicin plus AgomiR-223 vs. AgomiR-223 alone; ^&^*p* < 0.05, doxorubicin plus AgomiR-223 vs. doxorubicin alone). **c** Body weight of mice in the control (red), AgomiR-223 (orange), doxorubicin (green), or doxorubicin plus AgomiR-223 (violet) groups (***p < 0.001, control vs. doxorubicin alone; ^###^*p* < 0.001, doxorubicin plus AgomiR-223 vs. AgomiR-223 alone)

To investigate the in vivo effects of doxorubicin and miR-223 combined therapy for HCC, xenograft models were established via subcutaneous injection of HuH-7 cells. There was no significant difference in inhibition of tumor growth between the AgomiR-223 alone and the control group, while combined treatment resulted in notably increased inhibition of tumor growth compared with doxorubicin alone (Fig. 8a, b). Mouse body weight was measured following two weeks of chemotherapy; compared with the doxorubicin alone group, the weight of mice in the combined treatment group was higher although the difference did not achieve statistical significance (Fig. 8c). Taken together, these data suggest that miR-223 overexpression enhances the in vivo efficacy of doxorubicin for HCC and simultaneously reduces side effects.

## Discussion

Doxorubicin is commonly used for HCC chemoembolization, but its therapeutic benefits are usually limited by acquired resistance^2,3^. Combining autophagy-targeted agents with the traditional doxorubicin is regarded as a promising strategy to improve drug response^8–10^. miR-223 regulates many important cellular processes including multidrug resistance in HCC, and recent research indicates that miR-223 also suppresses excessive autophagy^18–24^. However, its role in modulating doxorubicin-induced autophagy in HCC cells remains unclear. In addition, although FOXO3a is reported to be suppressed by miR-223 and able to guide the doxorubicin-induced autophagy^10,29–32^, whether miR-223 might modulate autophagy via FOXO3a in HCC cells is not fully understood. This study revealed that miR-223 overexpression could enhance the therapeutic effect of doxorubicin for HCC in vitro and in vivo, and the underlying mechanism is through suppression of doxorubicin-induced autophagy via direct targeting of FOXO3a.

Autophagy, a catabolic process for self-protection, is central to adaptation to cellular stress; pharmacological or genetic inhibition of autophagy usually accelerates the demise of cells facing various challenges^7^. Doxorubicin treatment may easily trigger inducible autophagy and thereby results in intracellular drug degradation, chemoresistance, and malignant progression of cancer cells^8–10^. Our study also found that doxorubicin activated autophagy in HCC cells (Fig. 2a), while autophagy inhibition by chloroquine potentiates doxorubicin sensitivity (Fig. 2b), which is consistent with results reported elsewhere^11–13^. However, although autophagy has attracted much attention as a target to sensitize HCC cells to doxorubicin, such efforts have not yet generated clinically viable interventions.

The miRNA-based therapeutics possess excellent clinical potential in liver diseases. Insights into the role of miRNAs in doxorubicin-induced autophagy have made miRNAs attractive targets for HCC therapy; previously published studies demonstrated that miR-26 and miR-101 intervention could be used as a combined target reagent to enhance doxorubicin sensitivity in HCC cells via autophagy modulation^35,36^. In this study, we verified that miR-223 was expressed at low levels in HCC tissue, and that its overexpression sensitized HCC cells to doxorubicin (Fig. 1c–g), which is consistent with the findings of Zheng, Yang, and colleagues^22,23^. More importantly, although miR-223 was a well-identified miRNA in HCC^18–21^ and reported as an autophagy suppressor in cardiomyocytes^24^, we discovered for the first time its role in directing doxorubicin-induced autophagy in HCC cells, and specifically that miR-223 overexpression represses doxorubicin-induced autophagy in HCC cells at both steps of autophagosome and autolysosome formation (Fig. 3c–f). In addition, we demonstrated that autophagy is the critical underlying mechanism by which miR-223 influences doxorubicin resistance in HCC cells (Fig. 4a, b). These results establish that miR-223 intervention might be an effective approach for autophagy interference to reverse doxorubicin resistance. However, although we identified miR-223 as a suppressor of doxorubicin resistance, the mechanism by which HCC cells facing doxorubicin treatment establish a low expression of miR-223 to trigger resistance is still not clear and needs further exploration.

FOXO3a is a transcription factor which has been recently reported as a key regulator of autophagy^26–28^, while our previously published study demonstrated that FOXO3a was involved in the doxorubicin resistance of HCC cells via inhibition of epithelial-mesenchymal transition^33^. As shown in Fig. 6, FOXO3a was also discovered to influence doxorubicin sensitivity of HCC cells and block autophagy flux at both steps of autophagosome and autolysosome formation. These results are consistent with Salcher and Liu’s study^10,29^. Associated studies have reported that FOXO3a is a direct downstream target of miR-223 in multiple diseases^30–32^, but the role of FOXO3a in miR-223 regulated autophagy has never been elucidated. In this study we confirmed first the association between miR-223 and FOXO3a expression in HCC cells, and then found that FOXO3a plays critical roles in miR-223 related doxorubicin autophagy and resistance (Figs. 5 and 7). Other studies have reported that FOXO3a could regulate doxorubicin-induced autophagy via protein transcription including DEEP, ATG4B, and LC3^10,29^, hence further investigation is needed to clarify the in-depth mechanism by which FOXO3a directs miR-223-associated doxorubicin-induced autophagy in HCC.

Finally, we verified the enhancement of doxorubicin sensitivity by miR-223 overexpression in xenograft models, and that the combination of doxorubicin and agomiR-223 exerted a better therapeutic effect on tumor growth of HCC without negatively affecting body weight (Fig. 8). These results provide further support for the application of miR-223 co-intervention in prospective doxorubicin therapy. Nevertheless, many issues must be resolved in order to translate the strategy of altering miR-223 expression in HCC cells to clinical practice of doxorubicin use, including successfully reaching the local tumor tissues, specifically targeting the HCC cells, and effectively avoiding renal clearance and serum degradation. Despite these challenges, we are confident that many of these obstacles can be bypassed upon the development of new miRNA delivery systems which can specifically and effectively transport miR-223 into HCC cells and drastically raise doxorubicin sensitivity of HCC cells both in vitro and in vivo.

In summary, we developed a novel miRNA-based approach for autophagy interference, which allowed us to reverse the doxorubicin resistance for future chemotherapy against human HCC. In addition, we proved that FOXO3a mediated the regulation of miR-223 on doxorubicin-induced autophagy in HCC cells. Yet, the internal mechanisms by which, doxorubicin-treated HCC cells establish a low miR-223 expression to trigger resistance, and FOXO3a directs miR-223-associated doxorubicin-induced autophagy in HCC, are both unclear. Meanwhile, an optimized mode of miR-223 overexpression for clinical application warrants further investigation.

## Supplementary information

Supplementary Figure Legends

Supplementary Tables

Supplementary Figure S1

Supplementary Figure S2

Supplementary Figure S3

Supplementary Figure S4

Supplementary Figure S5

Supplementary Figure S6
