# Supplementary material for: miR-223 overexpression inhibits doxorubicin-induced autophagy by targeting FOXO3a and reverses chemoresistance in hepatocellular carcinoma cells
Source: Cell Death Dis. 2019 Nov 6;10(11):843. doi: 10.1038/s41419-019-2053-8 (PMC6834650; doi:10.1038/s41419-019-2053-8)
Supplement: Supplementary file 1 — Supplementary Figure Legends [file 41419_2019_2053_MOESM1_ESM.docx]

**Supplementary Figure Legends**

**Supplementary Figure S1.** **miR-223 overexpression sensitizes HCC cells to doxorubicin in vitro.** (A) Transfection efficacy of miR-223 mimic in different HCC cells (*** p<0.001, miR-223 mimic vs. NC mimic). (B) Transfection efficacy of miR-223 inhibitor in different HCC cells (** p<0.01, *** p<0.001, miR-223 inhibitor vs. NC inhibitor). (C) IC50s values of doxorubicin in miR-223 mimic transfected HCC cells (** p<0.01, *** p<0.001, miR-223 mimic vs. NC mimic). (D) IC50s values of doxorubicin in miR-223 inhibitor transfected HCC cells (** p<0.01, *** p<0.001, miR-223 inhibitor vs. NC inhibitor).

**Supplementary Figure S2. Autophagy inhibition potentiates doxorubicin sensitivity for HCC cells.** (A) Relative cell viability for HepG2, HuH-7, SNU-387 and SNU-449 cells treated with chloroquine (20 μM) for 48 h (ns p>0.05, Control vs. Chloroquine). (B) IC50s values of doxorubicin in HCC cells in the presence or absence of chloroquine (** p<0.01, *** p<0.001, Doxorubicin vs. Doxorubicin plus chloroquine).

**Supplementary Figure S3.** **miR-223 inhibitor promotes doxorubicin-induced autophagy by** **LC3-dual-fluorescence assay.** Up, mRFP-GFP-LC3 stable HepG2, HuH-7, SNU-387 and SNU-449 cells in the untreated groups, or groups with NC inhibitor or miR-223 inhibitor transfection following by doxorubicin treatment for 48 h were visualized by confocal microscopy. Down, number of GFP^+^/mRFP^+^-LC3 (yellow) and GFP^−^/mRFP^+^-LC3 (red) dots was scored on 50 cells. (^$$$^p<0.001, ***p<0.001, Control vs. NC inhibitor plus doxorubicin treatment; ^@@@^p<0.001, ns p>0.05, NC inhibitor plus doxorubicin treatment vs. miR-223 inhibitor plus doxorubicin treatment).

**Supplementary Figure S4.** **miR-223 inhibitor promotes doxorubicin-induced autophagy by** **TEM** **analysis.** Intracellular double-membrane vesicles in the ultrastructural feature of control HCC cells, and HCC cells transfected with NC inhibitor or miR-223 inhibitor following by doxorubicin treatment counterstained with 4% uranyl acetate observed by TEM. Scale bar = 10 μm.

**Supplementary Figure S5. FOXO3a play critical roles in doxorubicin-induced autophagy and chemo-sensitivity of HCC cells.** (A) FOXO3a protein expression of FOXO3a siRNA or NC siRNA transfected HCC cells. (B) FOXO3a mRNA expression of FOXO3a siRNA or NC siRNA transfected HCC cells. (C) IC50s values of doxorubicin in FOXO3a siRNA transfected HCC cells (** p<0.01, FOXO3a siRNA vs. NC siRNA).

**Supplementary Figure S6. FOXO3a expression in clinical samples and its correlation with overall survival of HCC patients.** (A) FOXO3a expression level of 374 HCC and 50 normal samples (p=0.088, Cancer samples vs. Normal samples). (B) Overall survival of HCC patients with high or low FOXO3a expression levels estimated using the Kaplan–Meier method and compared by the log-rank test (p = 0.22, low FOXO3a expression vs. high FOXO3a expression; Hazard Ratio = 1.24).
