# Supplementary material for: miR-223 overexpression inhibits doxorubicin-induced autophagy by targeting FOXO3a and reverses chemoresistance in hepatocellular carcinoma cells
Source: Cell Death Dis. 2019 Nov 6;10(11):843. doi: 10.1038/s41419-019-2053-8 (PMC6834650; doi:10.1038/s41419-019-2053-8)
Supplement: Supplementary file 2 — Supplementary Tables [file 41419_2019_2053_MOESM2_ESM.docx]

**Supplemental Tables**

**Supplemental Table S1. List of mimic, inhibitor and siRNA sequences used in this study.**

| **Name** |  | **Sequences (5’>3’ )** |
| --- | --- | --- |
| miR-223 mimic |  | Forward UAGCACCAUCUGAAAUCGGUUA |
|  |  | Reverse ACCGAUUUCAGAUGGUGCUAUU |
| miR-223 inhibitor |  | UGGGUAUUUGACAAACUGACA |
| NC siRNA |  | UUCUCCGAACGUGUCACGUTT |
| FOXO3A siRNA |  | ACUCCGGGUCCAGCUCCAC |

**Supplemental Table S2. List of primer sequences used in this study.**

| **Name** |  | **Forward Primer (5’>3’ )** |  | **Reverse Primer (5’>3’)** |
| --- | --- | --- | --- | --- |
| miR-223-3p |  | AGCTGGTGTTGTGAATCAGGCCG |  | TGGTGTCGTGGAGTCG |
| U6 |  | CTCGCTTCGGCAGCACA |  | AACGCTTCACGAATTTGCGT |
| FOXO3a |  | TGCGTGCCCTACTTCAAGGATAA |  | ACAGGTTGTGCCGGATGGA |
| GAPDH |  | ATCATCAGCAATGCCTCC |  | TCCTTCCACGATACCAAAG |
