# Supplementary figures and images for: miR-223 overexpression inhibits doxorubicin-induced autophagy by targeting FOXO3a and reverses chemoresistance in hepatocellular carcinoma cells
Source: Cell Death Dis. 2019 Nov 6;10(11):843. doi: 10.1038/s41419-019-2053-8 (PMC6834650; doi:10.1038/s41419-019-2053-8)

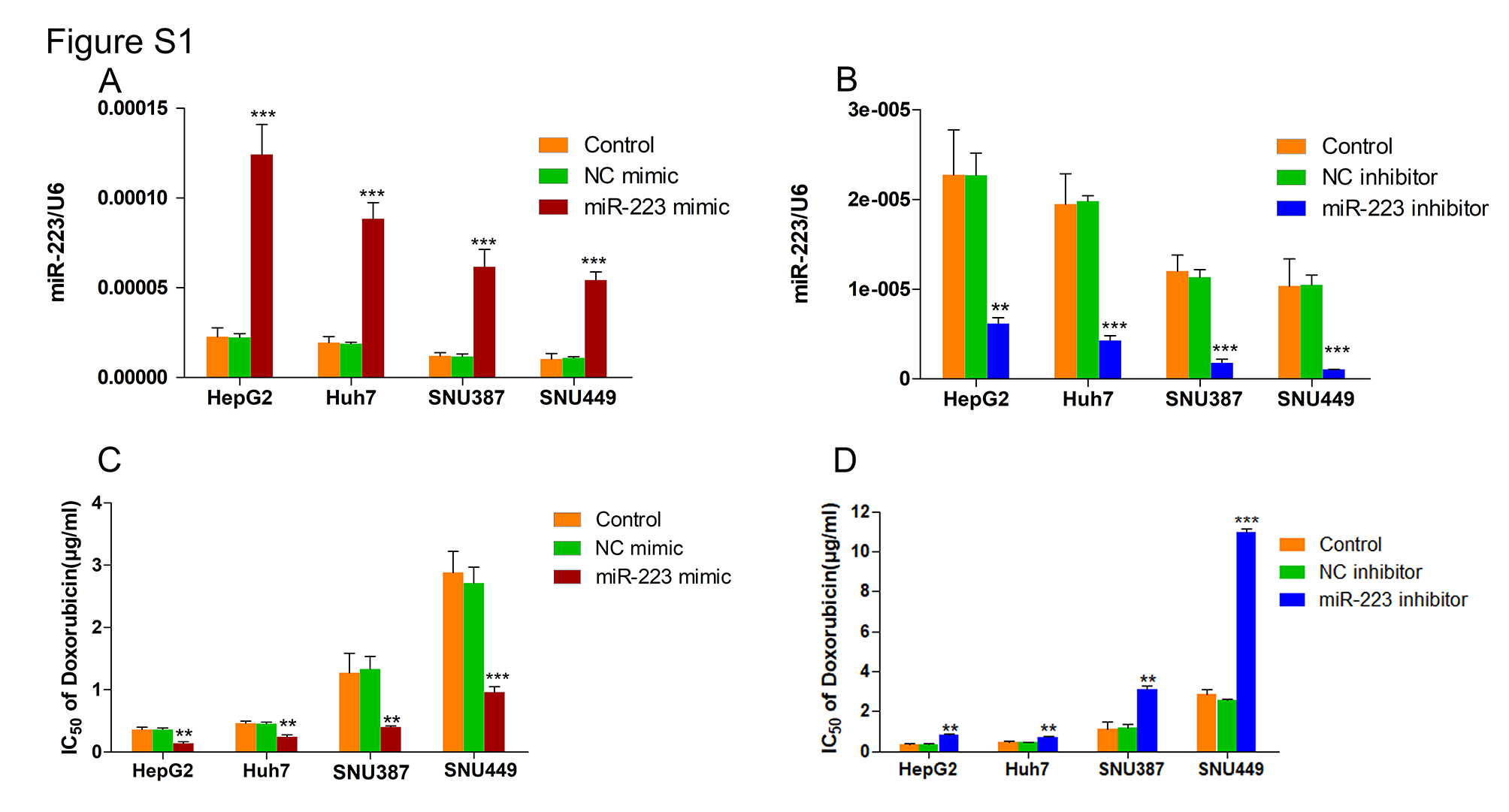

Supplement: Supplementary file 3 — Supplementary Figure S1 [file 41419_2019_2053_MOESM3_ESM.tif]

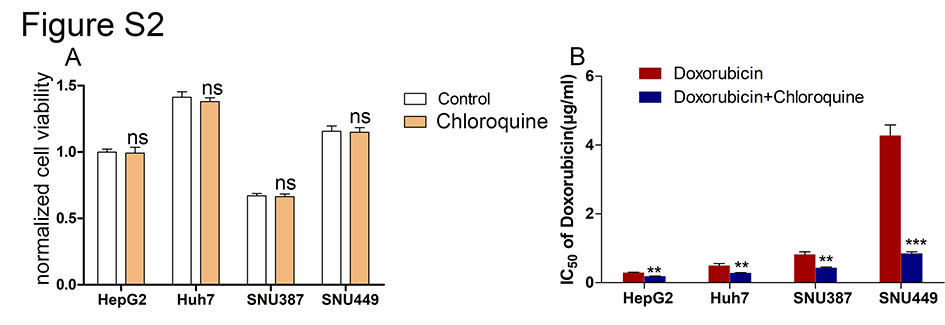

Supplement: Supplementary file 4 — Supplementary Figure S2 [file 41419_2019_2053_MOESM4_ESM.tif]

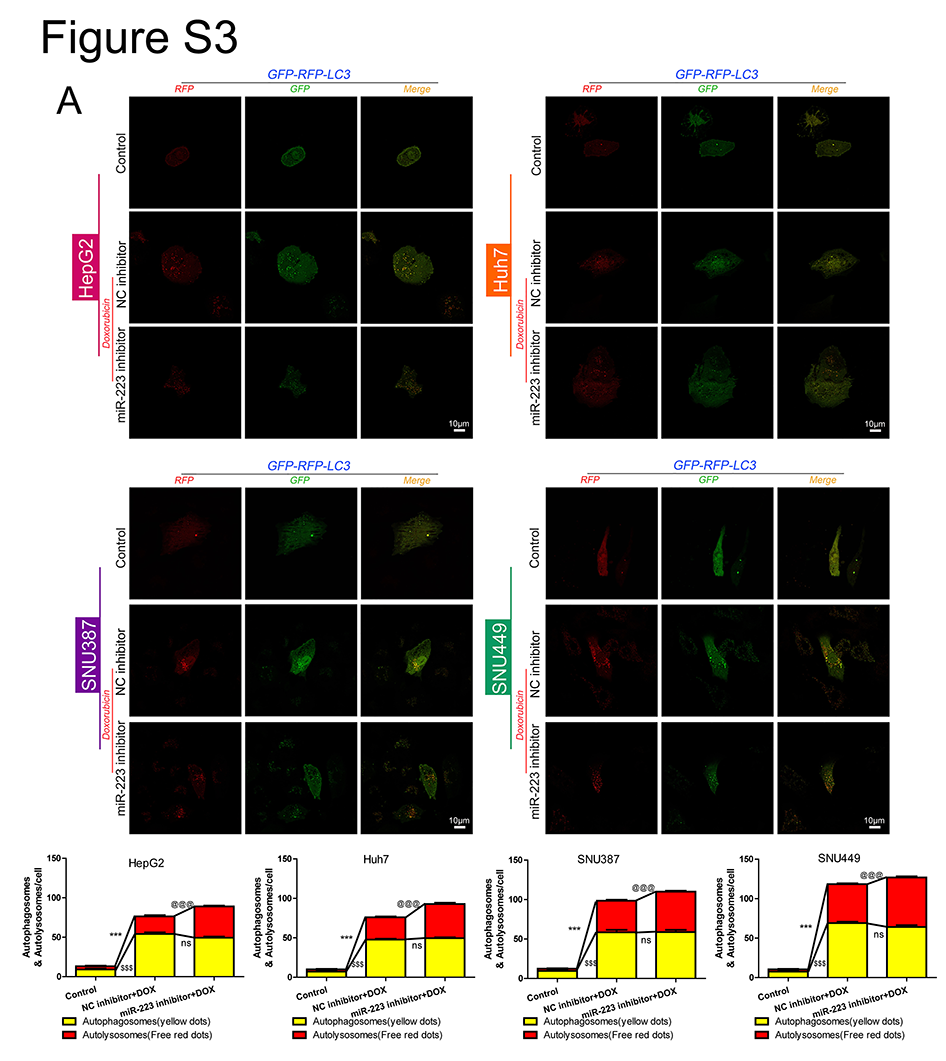

Supplement: Supplementary file 5 — Supplementary Figure S3 [file 41419_2019_2053_MOESM5_ESM.tif]

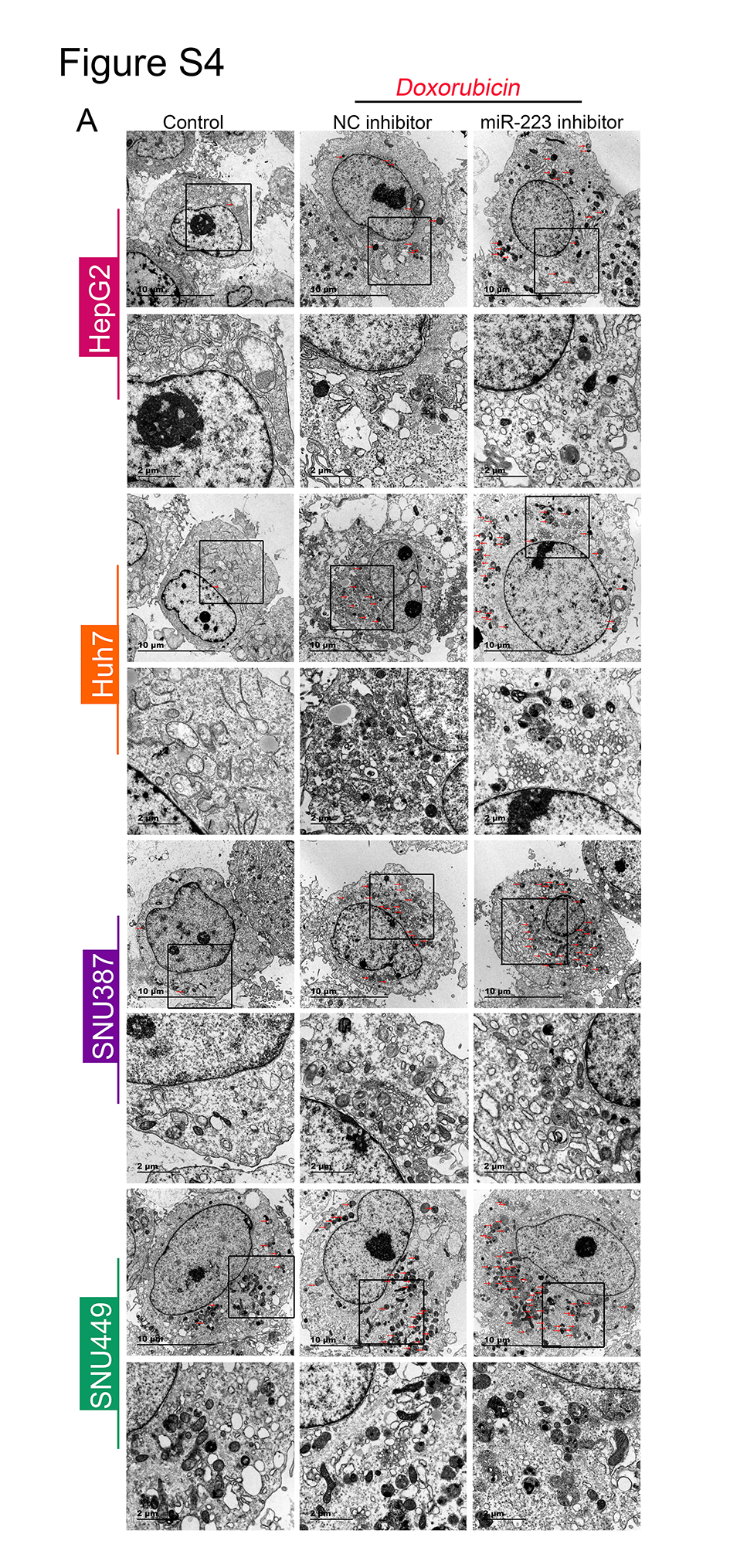

Supplement: Supplementary file 6 — Supplementary Figure S4 [file 41419_2019_2053_MOESM6_ESM.tif]

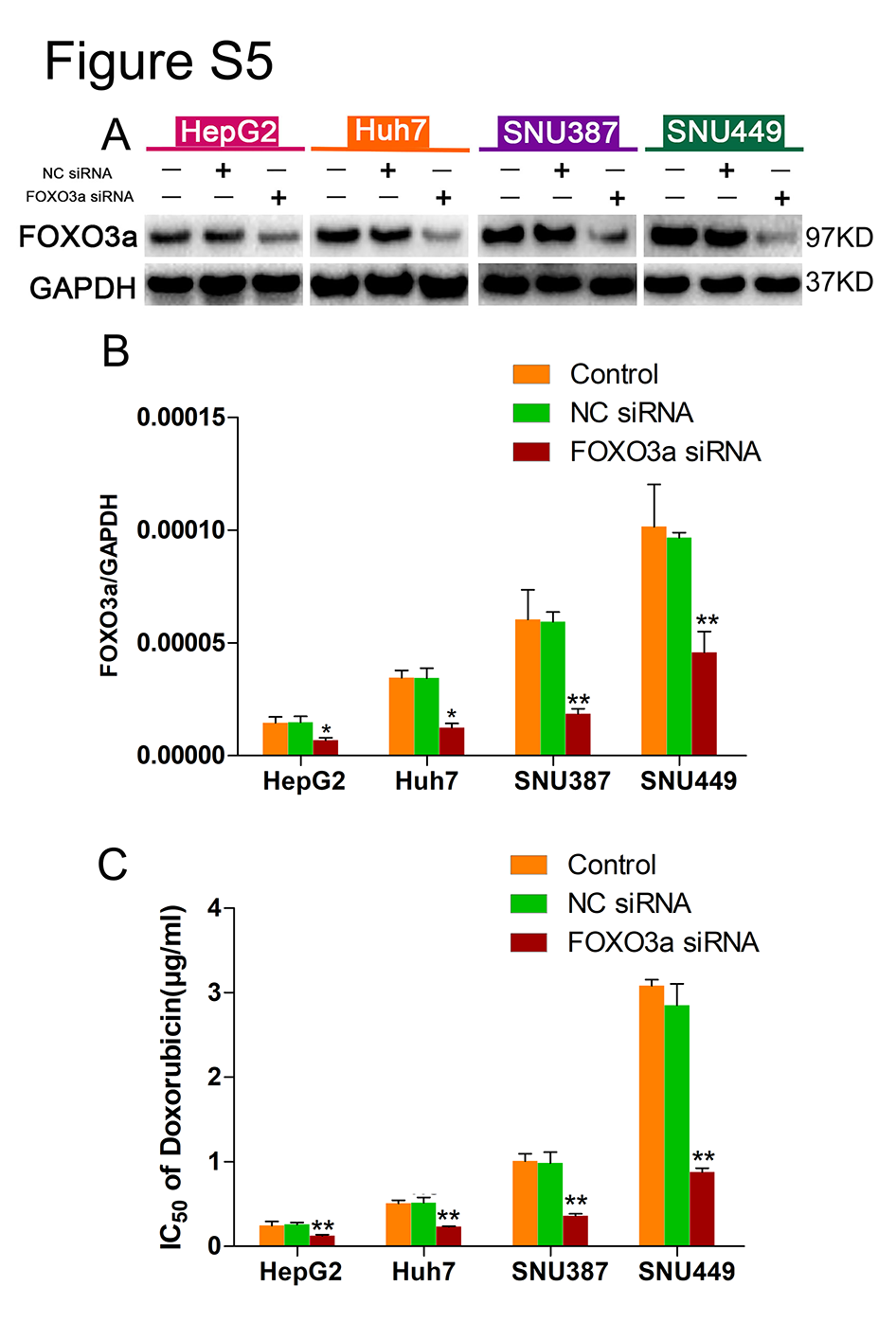

Supplement: Supplementary file 7 — Supplementary Figure S5 [file 41419_2019_2053_MOESM7_ESM.tif]

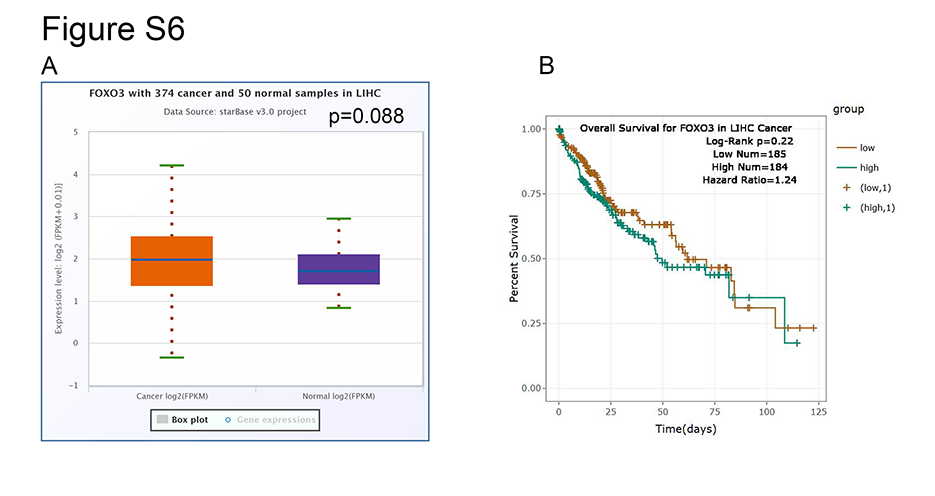

Supplement: Supplementary file 8 — Supplementary Figure S6 [file 41419_2019_2053_MOESM8_ESM.tif]
